# Supplementary material for: CDK6 inhibits de novo lipogenesis in white adipose tissues but not in the liver
Source: Nat Commun. 2024 Feb 5;15:1091. doi: 10.1038/s41467-024-45294-z (PMC10844593; doi:10.1038/s41467-024-45294-z)
Supplement: Supplementary file 1 — Supplementary Information [file 41467_2024_45294_MOESM1_ESM.pdf]

Supplementary Figures

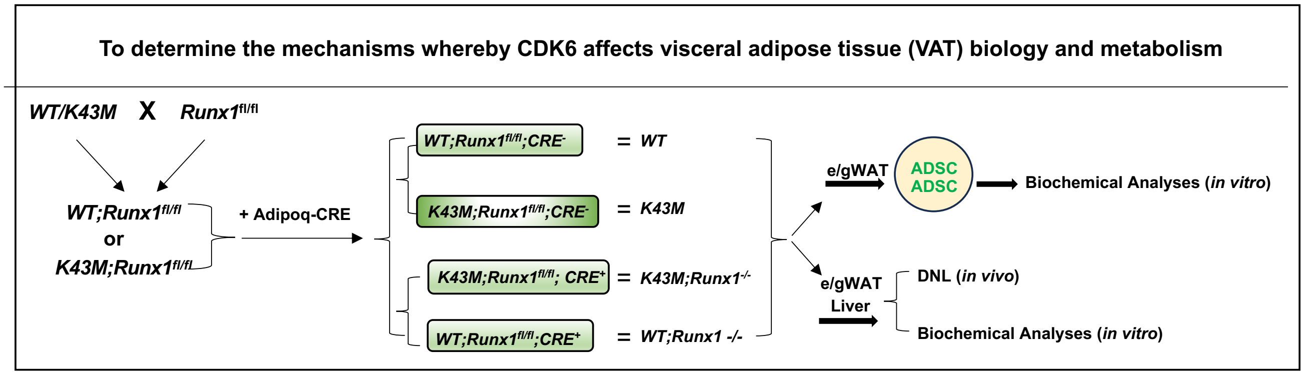

Supplementary Figure 1: Schematic summarizing the experimental strategies to determine the role of CDK6 on negative regulation of DNL in VAT but not in the liver.

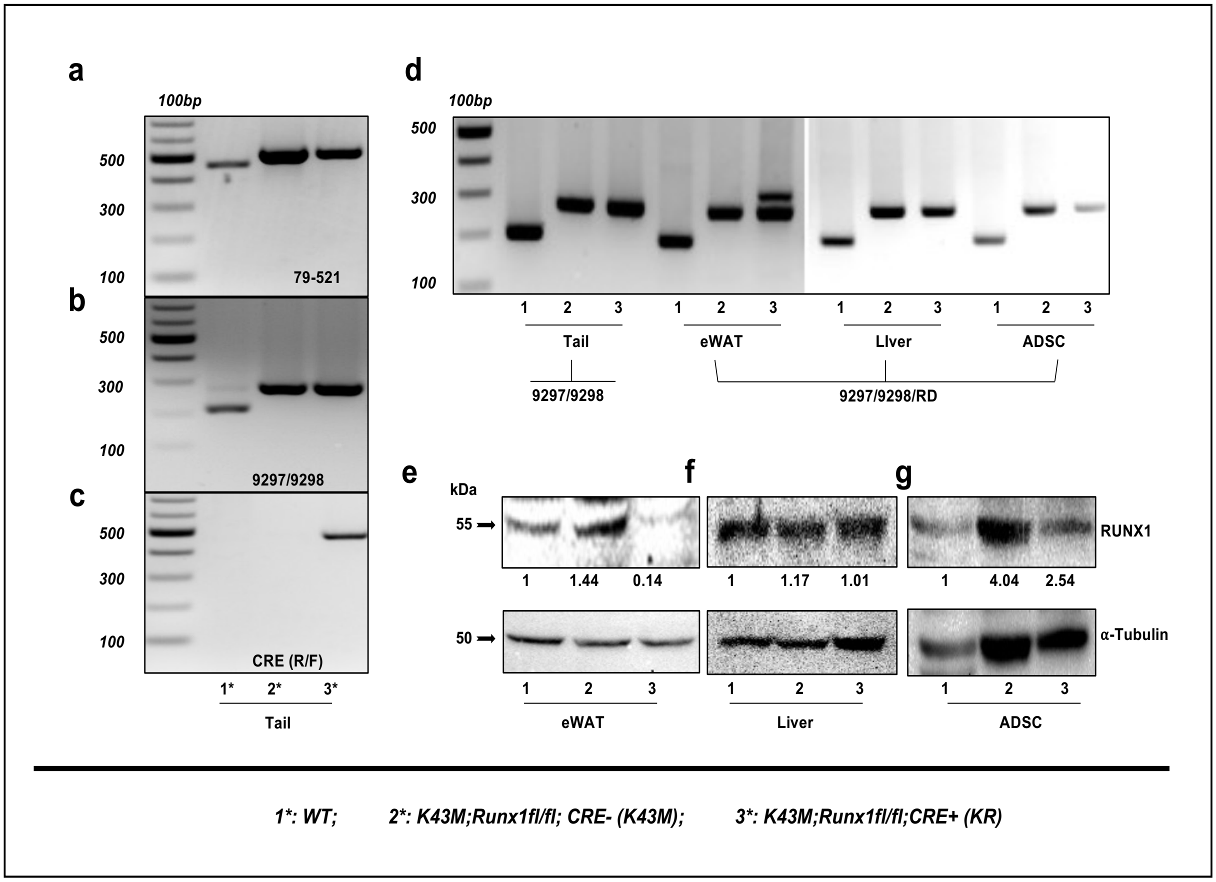

Supplementary Figure 2: PCR-based genotyping confirmed DNA recombination in the mature adipose tissues but not in the liver or in ADSCs.

(**a**)*WT* alleles (~466bp) and the deletion of LSL (*K43M*) by using primers "79" and "521" as indicated in previous studies[1,2] (with one 34 bp loxp site in the intron 1, ~500 bp for *K43M*); (**b**) *WT* (~203 bp) and Floxed *Runx1* alleles (~275 bp)[3]; (**c**) CRE expression (~500 bp)[4]; (**d**) Deleted Floxed Runx1 alleles (~310 bp)[3] on *KR* of eWAT but not on liver or ADSCs. (**e-g**) Representative immunoblots of the indicated protein levels in eWAT from 100 μg of cell lysates of male *WT, K43M, and KR* mice at 18 weeks of age. α-tubulin was used as internal loading control. The intensity of each protein was measured by **FluorChem M** system and then normalized to its internal control α-tubulin. Numbers shown below each protein are fold changes relative to *WT* control, which is arbitrarily set to 1 unit. Three independent experiments were repeated with similar results.

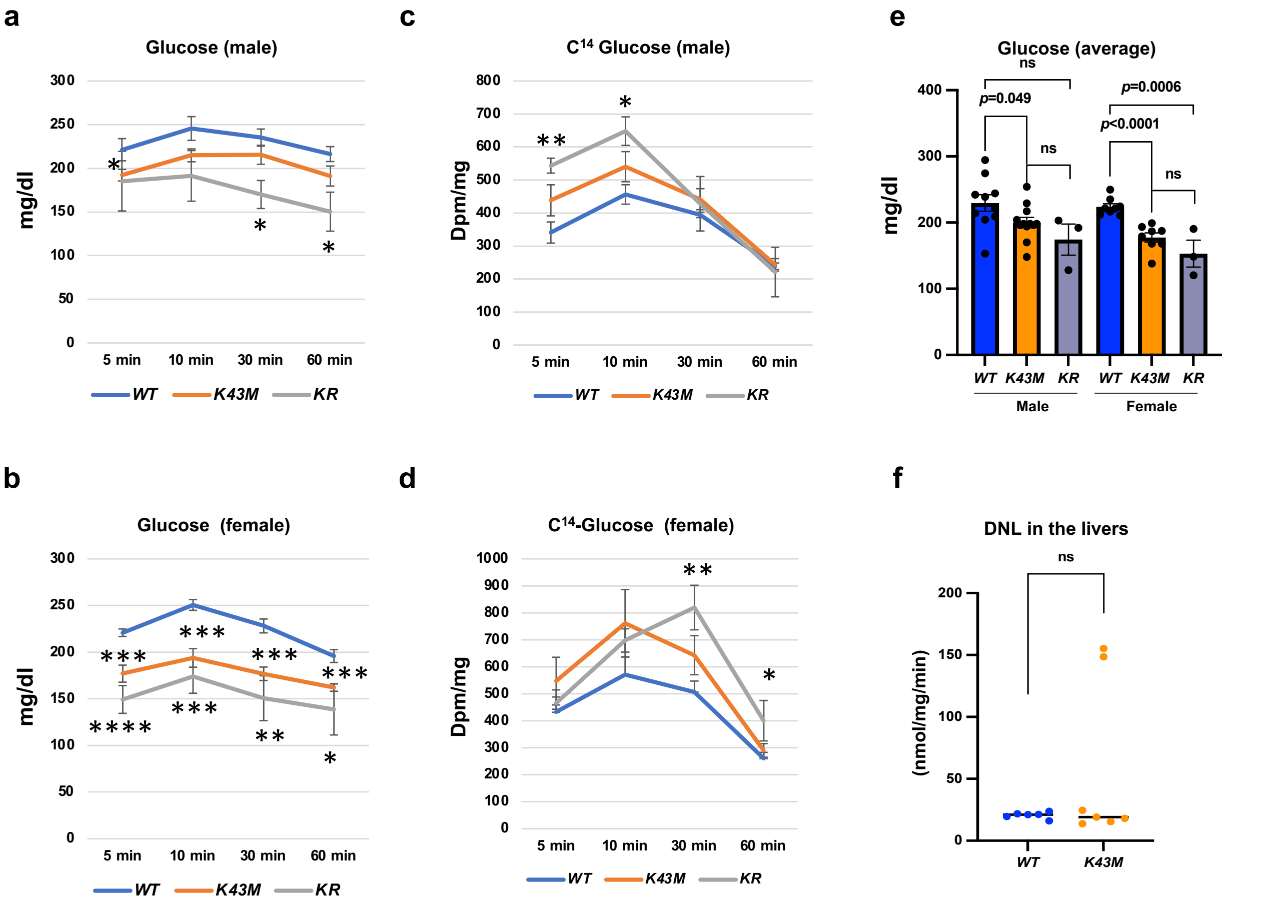

Supplementary Figure 3: *K43M* mice have increased DNL in VAT but not in the liver*.* (**a, b**): profiles of glucose concentrations at 5-, 10-, 30-, and 60-minutes post injection in male (**a**)**,** and female (**b**) mice. (**c, d*)***: profiles of D-(U-^{14}C)-glucose concentration obtained in male (**c**) and female (**d**) mice at 5-, 10-, 30-, and 60-minutes post injection. (**e**) Bar graphs summarized the average of glucose counts from 4 different time points obtained from (a) and (b). Data were shown as mean± S.E (n=10 and 8 for *WT* male and female, respectively; n=12 and 8 for *K43M* male and female, respectively; n=3 for *KM* male or female). (**f**) Scatter plot summarized the levels of D-(U-^{14}C)-glucose incorporation into TG of livers from *WT* and *K43M* mice. Data were shown as mean ± SE (n= 6-7 per group). We calculated statistical significance using two-tailed Student's T-test, with p <0.05 considered significant. For **Supplementary**

Figure 3a-d, *, $p < 0.05$, **, $p < 0.01$, ***, $p < 0.001$, ****, $p < 0.0001$. NS indicates no significance between two groups. The p values were summarized in Source data file. For **Supplementary Figure 3e,** the p values were summarized above two compared groups. NS indicates no significance between two groups.

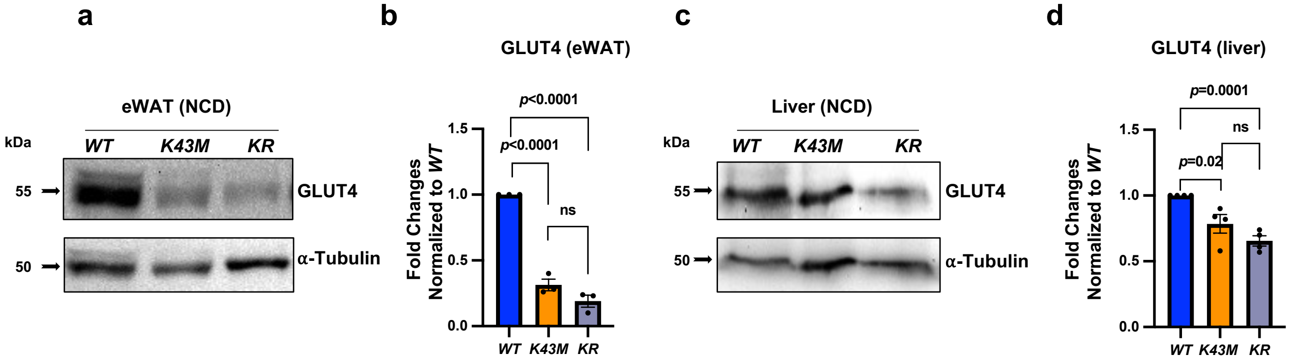

Supplementary Figure 4: GLUT4 protein was reduced in both eWAT and liver of *K43M* mice.

(**a, c**) Representative immunoblots of the indicated protein levels in eWAT or liver from 100 μg of cell lysates of male *WT, K43M, and KR* mice at the age of 18 weeks. α-tubulin was used as internal loading control. (**b, d**) Bar graphs summarized fold changes of different protein expression from 3-4 independent experiments. The intensity of each protein was measured by **FluorChem M** system and then normalized to its internal control α-tubulin. Protein fold changes were normalized to the control *WT,* which is arbitrarily set to 1 unit. Data were shown as mean ± S.E (n= 3-4 for different groups). We calculated statistical significance using two-tailed Student's T-test, with p <0.05 considered significant. For **Supplementary Figure 4b and d,** the p values were summarized above two compared groups. NS indicates no significance between two groups.

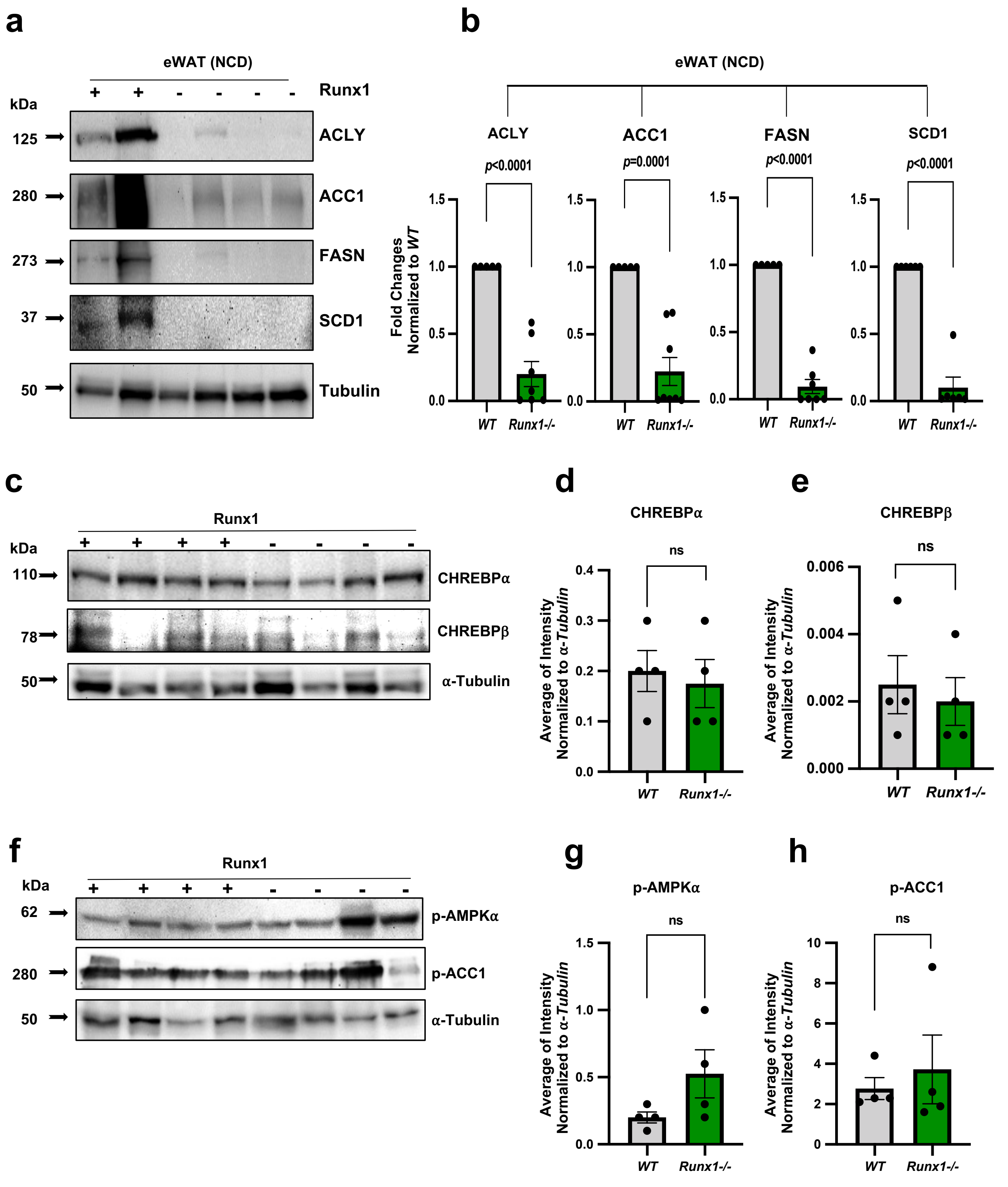

Supplementary Figure 5: Loss of *Runx1* on *WT* eWAT under NCD decreased protein levels of lipogenic enzymes but had no significant effects on levels of CHREBP and phosphorylation of

p-AMPKa and p-ACC1. (a, c, f) Representative immunoblots of the indicated protein levels in eWAT from 100 μg of cell lysates of male *WT, WT;Runx1$^{-/-}$* mice at the age of 18 weeks. α-tubulin was used as internal loading control. (**b, d, e, g, h**) Bar graphs summarized fold changes of different protein expression from 3-7 independent experiments. The intensity of each protein was measured by **FluorChem M** system and then normalized to its internal control α-tubulin. Protein fold changes were normalized to the control *WT,* which is arbitrarily set to 1 unit. Data were shown as mean ± S.E (n= 3-7 for different groups). We calculated statistical significance using two-tailed Student's T-test, with p <0.05 considered significant. For **Supplementary Figure 5b, d, e, g, and h,** the p values were summarized above two compared groups. NS indicates no significance between two groups.

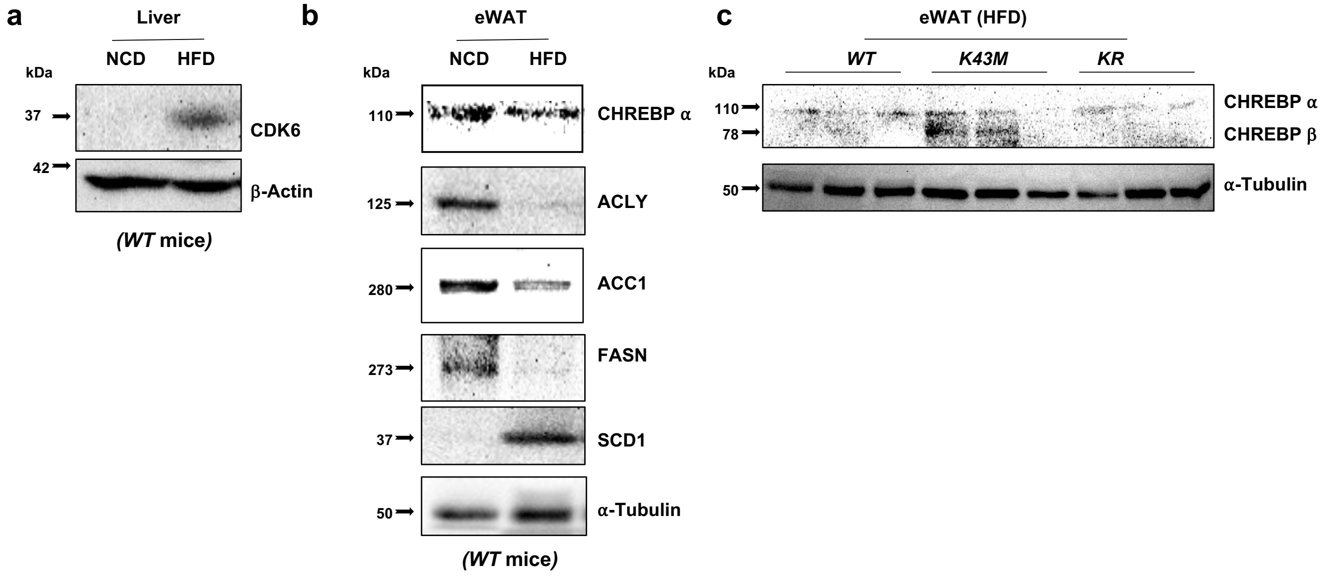

Supplementary Figure 6: CDK6 and DNL-associated proteins under HFD. (**a-c**) Representative immunoblots of the indicated protein levels in liver (**a**) or eWAT (**b, c**) from 100 μg of cell lysates of male *WT and K43M* mice under HFD for 14 weeks, starting at the age of 4 weeks. α-tubulin/β-Actin was used as loading control. Three independent experiments were repeated with similar results.

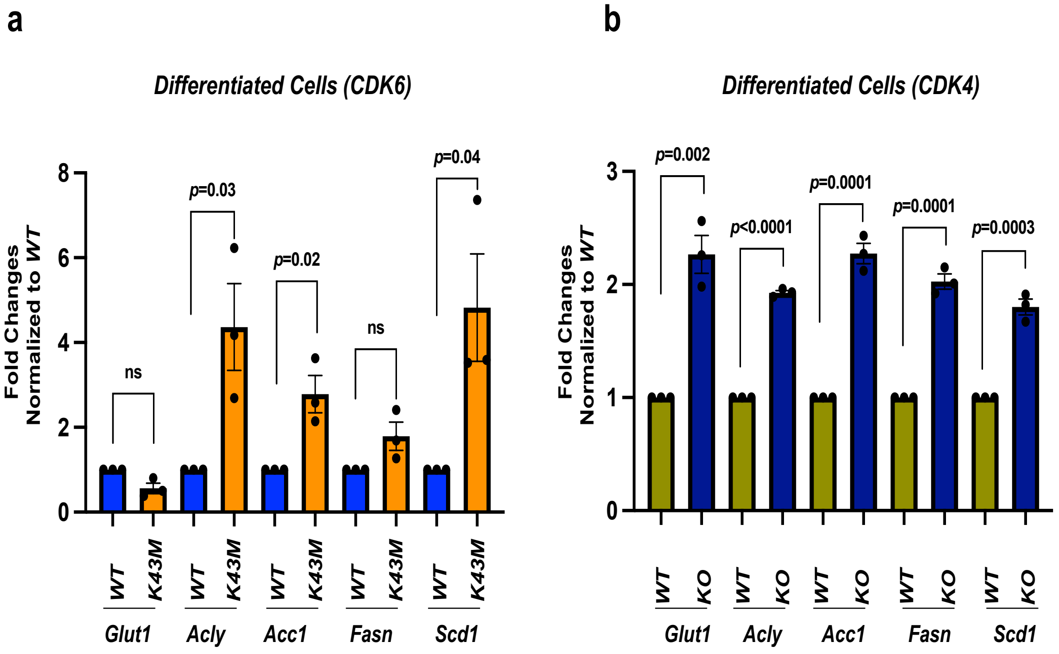

Supplementary Figure 7: Loss of CDK6 kinase activity or CDK4 protein increased expression of DNL-associated genes. Relative mRNA expression levels of *Glut1,* and DNL-specific markers (*Acly, Acc1, Fasn, and Scd1)* of differentiated cells derived from ADSCs of *WT and K43M* mice (**a**) or from MEFs of *WT and Cdk4-KO* mice (**b**) (n=3 for each group). The fold changes of each mRNA were normalized to the *WT* control*,* which was arbitrarily set to 1 unit. Data were shown as mean ± S.E (n=5 for WT/K43M, n=3 for WT/ *Cdk4-KO*). We calculated statistical significance using two-tailed Student's T-test, with $p < 0.05$ considered significant. The p values were summarized above two compared groups. NS indicates no significance between two groups.

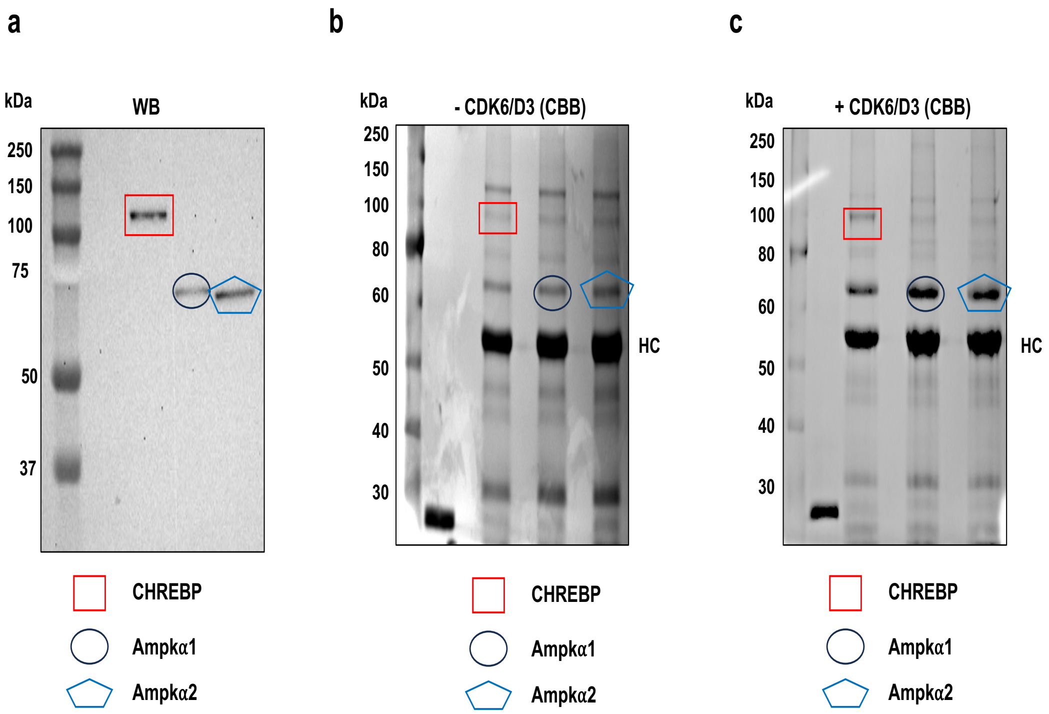

Supplementary Figure 8: CDK6/D3 phosphorylates CHREBP, AMPK-α1, and AMPK-α2. (a) immunoblot validation of overexpression lysates using anti-DDK antibody. Left, cell lysates from RC220626-transfected HEK293T cells (CHREBP); middle, cell lysates from RC218572-transfected HEK293T cells (AMPK-α1); right, cell lysates from RC210226-transfected HEK293T cells (AMPK-α2). (b, c) Coomassie Brilliant R-250 Blue (CBB) staining of SDS-page of purified fusion proteins (b) or after performing kinase assay with fusion proteins in the presence of CDK6/D3 (c). The visible stained proteins (~1μg) circled with different shapes were excised and submitted to proteomic analysis. The first lane next to protein ladder (b, c) is the 2.5μg of purified AMPK-α1 protein (~28KD) as a positive control for CBB staining. For **Supplementary Figure 8a-c,** three independent experiments were repeated with similar results.

a

Table 1:Total spectrum Count of phosphorylated peptides in the presence/absence of CDK6/D3

				Total Spectrum Count	Total Spectrum Count
				Human PO4	Human PO4
Accession Number	Alternate ID	Molecular Weight	Blank	CDK6/D3	No Kinase
Q9NP71	MLXIPL	93 kDa	0	431	67
Q13131	PRKAA1	64 kDa	0	454	31
P54646	PRKAA2	62 kDa	0	374	8

b

Table 2: Percent of phosphorylation in the presence/absence of CDK6/D3

CHREBP	CDK6/D3 Phosphorylation (%)	No Kinase Phosphorylation (%)	AMPKa1	CDK6/D3 Phosphorylation (%)	No Kinase Phosphorylation (%)	AMPKa2	CDK6/D3 Phosphorylation (%)	No Kinase Phosphorylation (%)
S47	5.87	0	T32	0.2	0	T21	1.29	0
S59	13.58	0	S172	6.86	0	T172	4.55	0
S65	5.36	0	S187	5.24	0	S176	14.55	0
S71	4.22	0	S293	4.7	0	S501	3.17	0
S196	97	100	T355	0.52	0	S513	1.47	0
T335	43.78	0	S356	26	0	S515	0.83	0
S361	2.41	0	T382	3.41	0	T517	0.25	0
S369	0.27	0	S415	100	0			
S449	40.93	0	T488	100	0			
S516	4.08	0	S496	4.93	0			
T520	2.76	0						
S602	100	100						
S614	72.88	47						
S618	0	52.99						
S619	10.21	0						
S631	4.51	0						

Supplementary Figure 9: CDK6/D3 phosphorylates CHREBP, AMPK-α1, and AMPK-α2. (**a**, **Table 1):** Total spectrum Count of phosphorylated peptides in the presence/absence of CDK6/D3. (**b,**

Table 2): Percent of phosphorylation in the presence/absence of CDK6/D3. The percent of phosphorylation is presented as %: sum of Total Iron Current (TIC) for phosphorylated peptides / sum of TIC for non-phosphorylated + phosphorylated peptides.

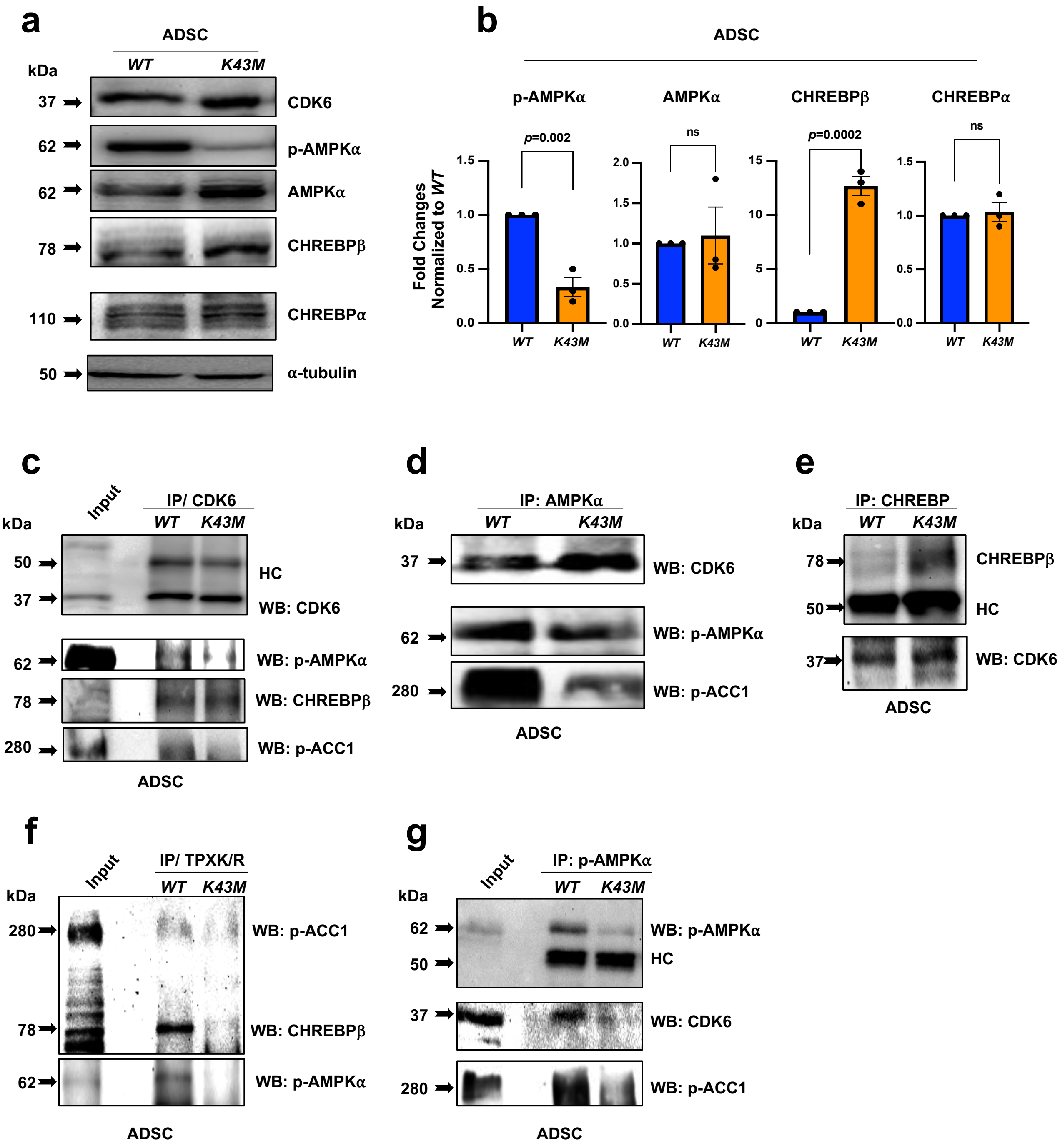

Supplementary Figure 10: ADSCs share some similarities, although not identical, to mature adipocytes (a) Representative immunoblots of the indicated protein levels from 100 μg of ADSCs isolated from eWAT of male *WT and K43M* mice at the age of 8 weeks under NCD and cultured in complete medium with high glucose (4.5 g/L). α-tubulin was used as internal loading control. The intensity of each protein was measured by FluorChem M system and then normalized to its internal

control α-tubulin. (**b**) Bar graphs summarized fold changes of different protein expression from 3 independent experiments. The intensity of each protein was measured by **FluorChem M** system and then normalized to its internal control α-tubulin. Protein fold changes were normalized to the control *WT,* which is arbitrarily set to 1 unit. Data were shown as mean ± S.E (n= 3 for different groups). We calculated statistical significance using two-tailed Student's T-test, with p <0.05 considered significant. The p values were summarized above two compared groups. NS indicates no significance between two groups. (**c-g**) IP-Westerns. CDK6, AMPKα**,** CHREBP, proteins containing CDK phosphorylated (p-T*PXK/R) consensus motifs, and p-AMPKα was immunoprecipitated from eWAT extracts derived from *WT* and *K43M* ADSCs and blotted with the indicated antibodies, HC indicates IgG heavy chain. Input represents 50 or 100 μg extracts from *WT* ADSCs.

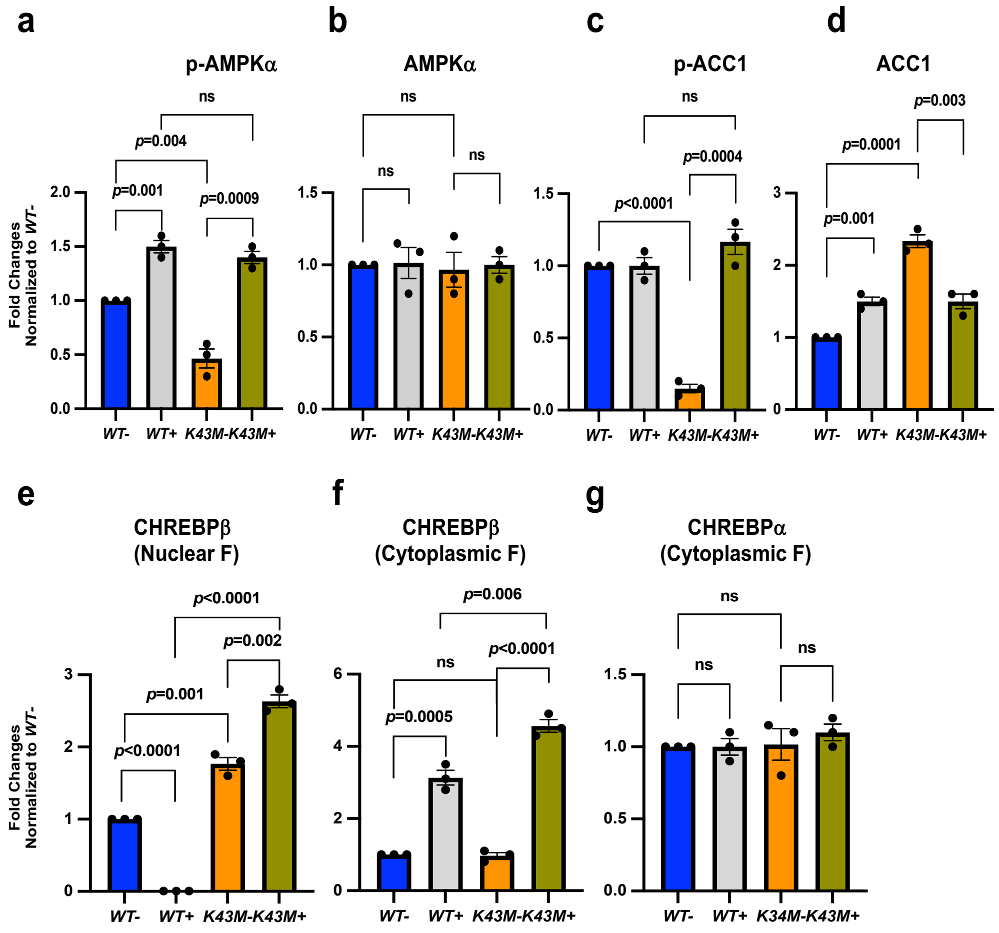

Supplementary Figure 11: Loss of CDK6 kinase activity leads to nuclear abundance of CHREBPβ. (**a-g**) Bar graphs summarized fold changes of different protein expression from 3 independent experiments. The intensity of each protein was measured by **FluorChem M** system and then normalized to its internal control α-tubulin. Protein fold changes were normalized to the control *WT-,* which is arbitrarily set to 1 unit. Data were shown as mean ± S.E (n= 3 for different groups). We calculated statistical significance using two-tailed Student's T-test, with p <0.05 considered significant. The p values were summarized above two compared groups. NS indicates no significance between two groups.

Supplementary Table 1, Primers sequences used for quantitative real-time PCR.

Gene	Forward Primers (5'-3')	Reverse Primers (5'-3')	References
Glut1	CTTCTCTGTCGGCCTCTTTGT	ACAGCTCCAAGATGGTGACCT	PNAS (2005) 102:1384-1389
ACLY	CTCACACGGAAGCTCCATAA	ACGCCCTCATAGACACCATC	Nature Communications (2020), 11, 575
ACC1	GTCCCCAGGGATGAACCAATA	GCCATGCTCAACCAAAGTAGC	Diabetes (2011) 60:464-76
FASN	GCTGCGGAAACTTCAGGAAAT	AGAGACGTGTCACTCCTGGACTT	J Biol Chem (2010) 285:25438-25447
SCD1	CCGGAGACCCCTTAGATCGA	TAGCCTGTAAAAGATTTCTGCAAA	PNAS (2005) 29:10297-10302
PGC-1α	TGCCCAGATCTTCCTGAACT	TCTGTGAGAACCGCTAGCAA	Nature (2008) 454:961-967
UCP-1	ACTGCCACACCTCCAGTCATT	CTTTGCCTCACTCAGGATTGG	Nature (2008) 454:961-967

36b4	AGATGCAGCAGATCCGCAT	GTTCTTGCCCATCAGCACC	Cell Metab (2008) 7: 485-495

References:

1. Hu, M.G., *et al.* A requirement for cyclin-dependent kinase 6 in thymocyte development and tumorigenesis. *Cancer Res* **69**, 810-818 (2009).
2. Hu, M.G., *et al.* CDK6 kinase activity is required for thymocyte development. *Blood* **117**, 6120-6131 (2011).
3. Chen, M.J., Yokomizo, T., Zeigler, B.M., Dzierzak, E. & Speck, N.A. Runx1 is required for the endothelial to haematopoietic cell transition but not thereafter. *Nature* **457**, 887-891 (2009).
4. Hou, X., *et al.* CDK6 inhibits white to beige fat transition by suppressing RUNX1. *Nat Commun* **9**, 1023 (2018).
