# Supplementary material for: CDK6 inhibits de novo lipogenesis in white adipose tissues but not in the liver
Source: Nat Commun. 2024 Feb 5;15:1091. doi: 10.1038/s41467-024-45294-z (PMC10844593; doi:10.1038/s41467-024-45294-z)
Supplement: Supplementary file 3 — Reporting Summary [file 41467_2024_45294_MOESM3_ESM.pdf]

nature portfolio

Corresponding author(s): Miaofen hu

Last updated by author(s): 01/12/2024

Reporting Summary

Nature Portfolio wishes to improve the reproducibility of the work that we publish. This form provides structure for consistency and transparency in reporting. For further information on Nature Portfolio policies, see our Editorial Policies and the Editorial Policy Checklist.

Statistics

For all statistical analyses, confirm that the following items are present in the figure legend, table legend, main text, or Methods section.

n/a	Confirmed	
☐	☒	The exact sample size (*n*) for each experimental group/condition, given as a discrete number and unit of measurement
☐	☒	A statement on whether measurements were taken from distinct samples or whether the same sample was measured repeatedly
☐	☒	The statistical test(s) used AND whether they are one- or two-sided<br>*Only common tests should be described solely by name; describe more complex techniques in the Methods section.*
☒	☐	A description of all covariates tested
☐	☒	A description of any assumptions or corrections, such as tests of normality and adjustment for multiple comparisons
☐	☒	A full description of the statistical parameters including central tendency (e.g. means) or other basic estimates (e.g. regression coefficient) AND variation (e.g. standard deviation) or associated estimates of uncertainty (e.g. confidence intervals)
☐	☒	For null hypothesis testing, the test statistic (e.g. *F*, *t*, *r*) with confidence intervals, effect sizes, degrees of freedom and *P* value noted<br>*Give P values as exact values whenever suitable.*
☒	☐	For Bayesian analysis, information on the choice of priors and Markov chain Monte Carlo settings
☒	☐	For hierarchical and complex designs, identification of the appropriate level for tests and full reporting of outcomes
☒	☐	Estimates of effect sizes (e.g. Cohen's *d*, Pearson's *r*), indicating how they were calculated

Our web collection on statistics for biologists contains articles on many of the points above.

Software and code

Policy information about availability of computer code

Data collection	No commercial or custom code was used to collect data in this study
Data analysis	GraphPad Prism 9 was used to generate bar graphs.<br>For comparisons of two experimental groups, unpaired two-tailed Student’s t tests were employed (Excel).<br>For in vivo de novo lipogenesis, DNL in conscious mice was analyzed by using stable isotopes coupled to mass spectrometry analysis.<br>For phosphorylation analysis, Mass spectrometry analysis was used.<br>For intensity of protein was measured by FluorChem M system and normalized to alpha-tubulin or beta-actin.<br>For determining the gene expression, we used RT-PCR (Roche, light cyber@480)

For manuscripts utilizing custom algorithms or software that are central to the research but not yet described in published literature, software must be made available to editors and reviewers. We strongly encourage code deposition in a community repository (e.g. GitHub). See the Nature Portfolio guidelines for submitting code & software for further information.

Data

Policy information about availability of data

All manuscripts must include a data availability statement. This statement should provide the following information, where applicable:

- Accession codes, unique identifiers, or web links for publicly available datasets
- A description of any restrictions on data availability
- For clinical datasets or third party data, please ensure that the statement adheres to our policy

The Mass Spectrometry data (Raw and peak data) generated by Beth Israel Deaconess Medical Center (BIDMC) for this study are available at MassIVE with the identifier (doi:10.25345/C57D2QJ4C) (reference number: MassIVE MSV000093063. The result data generate by Dr. John M Asara from BIDMC are available at Figshare DOI: https://doi.org/10.6084/m9.figshare.24265774. All the data generated or analyzed during this study are included in this re-submitted manuscript and its supplementary information files.

Field-specific reporting

Please select the one below that is the best fit for your research. If you are not sure, read the appropriate sections before making your selection.

☒ Life sciences ☐ Behavioural & social sciences ☐ Ecological, evolutionary & environmental sciences

For a reference copy of the document with all sections, see nature.com/documents/nr-reporting-summary-flat.pdf

Life sciences study design

All studies must disclose on these points even when the disclosure is negative.

Sample size

No sample sizes were per-calculated. Biological and experimental replicates were determined empirically for particular experiments. The sample size will be chosen based on expected differences between experimental and control groups in order to provide adequate power to detect a significant difference specifying $\alpha = 0.05$, two-tailed testing, and power (= 1-β) of 80%, using commercially available software packages (Statistical Solutions nQuery Advisor; http://www.statsol.ie/nquery/nquery.htm). Although these conditions vary, our control cohorts are usually designed to have fairly tight incidence curves, allowing us to be adequately powered with experimental recipient cohorts of average size n =5-10.

Data exclusions

No data was excluded from analysis.

Replication

Reproducibility of experimental findings was verified by different investigator or by the same investigator at different time. The experiments were repeated at least three times. In our figure legends, we described the times for each experiment performed.

Randomization

To examine the effect of CDK6 Kinase activity on DNL, mice were randomly chosen for in vivo study among different genotypes.
To examine the effect of CDK6 Kinase activity on DNL in vitro, mice were randomly chosen to isolate adipose tissue derived stem cells for differentiation.
To determine if CDK4/6 duel inhibitor recapitulates the phenotypes observed in K43M mice under HFD, C57BL/6 mice were randomly grouped into two groups: Vehicle (0.5% methycellulose) and Lee (200 mg/kg daily).

We chose mice randomly but based on three different genotypes. That is both age- and sex- matched wild type mice with normal CDK6 protein, kinase inactive protein K43M mice, and K43M; Runx1-/- mice. We also used both male and female mice for all the experiments.

Blinding

In vivo de novo lipogenesis (DNL) was analyzed blindly in UMass Chan Medical School- Metabolic Disease Research Center by Dr. Jason, Kim's group.
The investigators are not possible to be blind for these experiments due to different strains of mice need to be sent and certain numbers of animals need to be done.

Reporting for specific materials, systems and methods

We require information from authors about some types of materials, experimental systems and methods used in many studies. Here, indicate whether each material, system or method listed is relevant to your study. If you are not sure if a list item applies to your research, read the appropriate section before selecting a response.

Materials & experimental systems

n/a	Involved in the study
☐	☒ Antibodies
☐	☒ Eukaryotic cell lines
☒	☐ Palaeontology and archaeology
☐	☒ Animals and other organisms
☒	☐ Human research participants
☒	☐ Clinical data
☒	☐ Dual use research of concern

Methods

n/a	Involved in the study
☒	☐ ChIP-seq
☒	☐ Flow cytometry
☒	☐ MRI-based neuroimaging

Antibodies

Antibodies used

Antibodies used in this study included CDK6 (C-21, Santa Cruz), RUNX1 (Ab23980, Rabbit polyclonal antibody, Abcam), alpha-Tubulin (Sigma, T6199), GLUT1 (12939S, Cell Signaling Technology-CST), GLUT4 (2213S, CST), ACLY (4332S, CST), FASN (8335T, CST), p-ACC1 (3661S, CST, S79), ACC1 (3676S, CST), SCD1 (2438S, CST), p-AMPK-alpha (2535S, CST, T172), AMPK-alpha(2603S, CST), CHREBP (58069S, CST), mTOR (2983S, CST), p-mTOR S2448 (5536S, CST), p-PXS*P (2325S, CST), and p-T*PXK/R (14371S, CST).

Validation

We performed co-immunoprecipitation assay to examine if CDK6 interacts with CHREBP and AMPKα. First, we ensured that the antibodies of CHREBP, CDK6, T*PXK/R, and AMPKα used were able to immunoprecipitate their respective antigens from the cell lysates. Normal rabbit IgG was used as the negative control. Figure 6a-d demonstrated that all the tested target proteins were able to bind their own antibodies but not to IgG, indicating target protein binds to its antibody specifically (Fig. 6a-d). Furthermore, we validated CHREBP antibody by knockdown CHREBP by ShRNA. All other antibodies used in the studies were validated by vendors. We have been cited their names and catalogs in Methods and described above as well.

All the antibodies used in this study are validated by companies listed above.

Eukaryotic cell lines

Policy information about cell lines

Cell line source(s)

293T cells

Authentication

Adipose-tissues derived stem cells from WT and K43M mice, WT and CDK4 deficient mouse embryonic fibroblasts (MEFs).

Mycoplasma contamination

None

Commonly misidentified lines
(See ICLAC register)

None

Animals and other organisms

Policy information about studies involving animals; ARRIVE guidelines recommended for reporting animal research

Laboratory animals

Different ages of WT (C57BL/6), K43M, K43M;Runx1-/-, WT;Runx1-/- were used in this study. We made K43M mice. C57BL/6, RUNX1fl/fl and Adiponectin-CRE mice were purchased from Jackson Lab.

Wild animals

None

Field-collected samples

None

Ethics oversight

All animal experiments were approved by Institutional Animal Care and Use Committee (IACUC) of Tufts University/Tufts Medical Center. All procedures were performed in accordance with protocols approved by IACUC of Tufts University/Tufts Medical Center.

Note that full information on the approval of the study protocol must also be provided in the manuscript.
